# N-linked glycosylation enzymes in the diatom *Thalassiosira oceanica* exhibit a diel cycle in transcript abundance and favor for NXT-type sites

**DOI:** 10.1038/s41598-021-82545-1

**Published:** 2021-02-05

**Authors:** Joerg Behnke, Alejandro M. Cohen, Julie LaRoche

**Affiliations:** 1 Department of Biology, Life Science Centre, Dalhousie University, 1355 Oxford Street, PO BOX 15000, Halifax, NS B3H 4R2 Canada; 2 Department of Biochemistry and Molecular Biology, Life Science Research Institute, Dalhousie University, 1344 Summer Street, PO Box 15000, Halifax, NS B3H 4R2 Canada

**Keywords:** Cell biology, Molecular biology, Plant sciences, Ocean sciences

## Abstract

N-linked glycosylation is a posttranslational modification affecting protein folding and function. The N-linked glycosylation pathway in algae is poorly characterized, and further knowledge is needed to understand the cell biology of algae and the evolution of N-linked glycosylation. This study investigated the N-linked glycosylation pathway in *Thalassiosira oceanica*, an open ocean diatom adapted to survive at growth-limiting iron concentrations. Here we identified and annotated the genes coding for the essential enzymes involved in the N-linked glycosylation pathway of *T. oceanica*. Transcript levels for genes coding for calreticulin, oligosaccharyltransferase (OST), N-acetylglucosaminyltransferase (GnT1), and UDP-glucose glucosyltransferase (UGGT) under high- and low-iron growth conditions revealed diel transcription patterns with a significant decrease of calreticulin and OST transcripts under iron-limitation. Solid-phase extraction of N-linked glycosylated peptides (SPEG) revealed 118 N-linked glycosylated peptides from cells grown in high- and low-iron growth conditions. The identified peptides had 81% NXT-type motifs, with X being any amino acids except proline. The presence of N-linked glycosylation sites in the iron starvation-induced protein 1a (ISIP1a) confirmed its predicted topology, contributing to the biochemical characterization of ISIP1 proteins. Analysis of extensive oceanic gene databases showed a global distribution of calreticulin, OST, and UGGT, reinforcing the importance of glycosylation in microalgae.

## Introduction

Posttranslational modifications of proteins are essential for proper protein function in all living organisms, with N-linked glycosylation widely prevalent in numerous proteins. N-linked glycosylation is primarily used for protein folding control in the endoplasmic reticulum with additional functions based on the matured glycan structures in the Golgi apparatus. Whereas putative functions of N-linked glycosylation in algae are unknown, functions in plants are slightly better understood, and, for example, a complete blockage of N-linked glycosylation leads to embryonic death, demonstrating the importance of this process in plants^1,2^. The maturation of N-linked glycan structures differs based on specific enzymes in the Golgi apparatus, and while vertebrates show a high diversity of glycan structures, homologues for many of the enzymes present in vertebrates have not been identified in plants and microalgae^3,4^. The diversity of glycan structures in vertebrates is reflected in functions ranging from cell–cell communication, their involvement in auto-immune diseases, to inflammatory reactions^5^. Therefore, it is not surprising that N-linked glycosylation is often an essential component for the functionality of biopharmaceuticals, where microalgae are seen as an attractive alternative for recombinant protein production as they are eukaryotic, photo-autotroph and fast-growing^6^. The first production of a functional monoclonal antibody in *Phaeodactylum tricornutum* was a significant step towards a recombinant protein expression system in diatoms for pharmaceutical usage^7–10^. *P. tricornutum* is capable of N-linked glycosylation resulting mainly in high-mannose glycans^4^. The N-linked glycosylation pathway in eukaryotes is a multi-step process that starts in the endoplasmic reticulum (ER) and continues throughout the Golgi apparatus.

The first step is the formation of a lipid-linked oligosaccharide (LLO) by well-conserved asparagine-linked enzymes (ALG)^11^. An oligosaccharyltransferase (OST) attaches the LLO to an asparagine that is part of an NXS/T motif, with x being any amino acid except proline^11,12^. The attached glycan is then trimmed in the ER before N-linked glycosylated proteins enter a folding control in the ER. The exact mechanism of the folding control is unknown, but the chaperones calreticulin and calnexin are involved in the folding process, and UDP-glucose glucosyltransferase (UGGT) glycosylates misfolded proteins, forcing these proteins into a new folding control^13^. Correctly folded proteins enter the Golgi apparatus, while misfolded proteins are eventually exported out of the ER and degraded in the proteasome. Once a glycosylated protein reaches the Golgi-apparatus, the glycan is first trimmed and then either modified by N-acetylglucosaminyltransferase I (GnT1) for further glycan maturation by a variety of enzymes^14^ or enzymes alter the glycan structures in a GnT1-independent fashion^4^.

Here we report on the fully annotated complement of genes involved in the N-linked glycosylation pathway in the open ocean diatom *Thalassiosira oceanica* and confirm previous findings on the N-linked glycosylation pathway in the coastal diatom *P. tricornutum*^4^*.* Further, we identified 118 N-linked glycosylated peptides in *T. oceanica* and compared these results with other microalgae through analysis of previously published data from *Chlamydomonas reinhardtii*^15^ and *Botyrococcus braunii*^16^*.*

*T. oceanica* has been the focus of research efforts on diatoms because of its global ecological relevance and its ability to withstand very low concentrations of iron^17–20^. Vast regions in the ocean lack sufficient iron and iron-addition experiments promoted algae growth in the iron-deprived regions of the ocean^21^, demonstrating significant implications of iron on the global carbon budget as well as on nutrient fluxes in the ocean^22^. Besides the upregulation of high-affinity iron uptake proteins^19^, the substitution of iron-containing enzymes^23^, and the downregulation of chloroplast-related genes^17^, *T. oceanica* reveals a restructuring of the cell surface proteome under low-iron conditions^17^. The central role of N-linked glycosylation for cell surface proteins and the restructuring of the cell surface proteome led us to perform transcriptomic analyses of genes involved in the N-linked glycosylation pathway under high- and low-iron conditions as we anticipated changes in the N-linked glycosylation pathway on a transcriptional level under iron stress. In humans, N-linked glycosylation has important implications on the functionality of iron-regulated proteins. The transferrin receptor 2 (TFR2) exhibited N-linked glycosylations as necessary for its correct function^24^, and the proteasomal degradation of Zrt/IRT-like protein 14 (ZIP14) depends on its deglycosylation^25^. We analyzed transcript levels of three genes in *T. oceanica*, including OST, calreticulin, and UGGT, critical to the control of protein folding and the attachment of glycans to N-linked glycosylation sites in the ER. The discussion on GnT1-dependant glycan structures in microalgae^4,16,26^ led us to analyze expression levels of GnT1.

## Results

### Identification of enzymes involved in N-linked glycosylation

**Figure 1:**
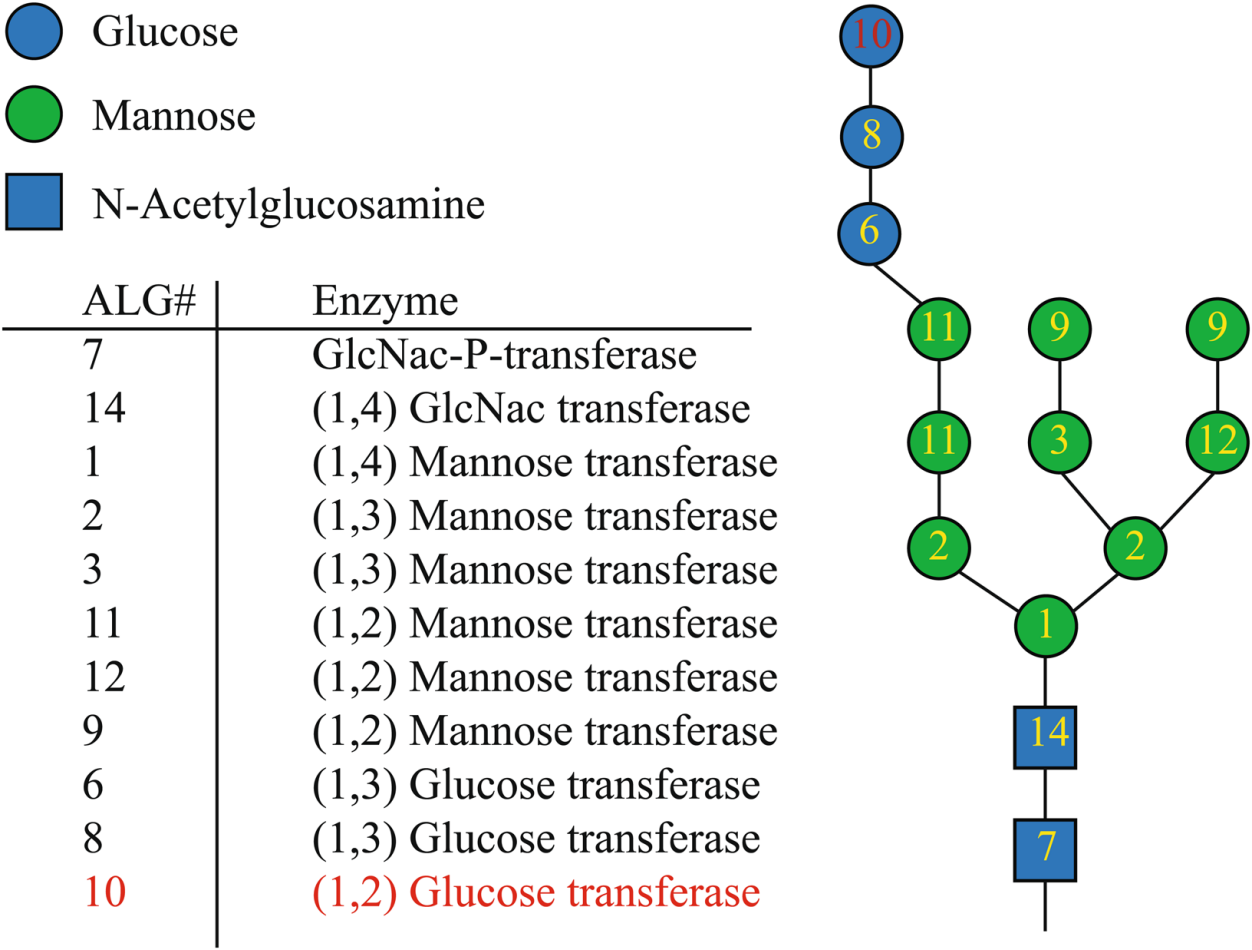
Overview of asparagine-linked glycosylation (ALG) enzymes and the lipid-linked glycan structure. The structure of a lipid-linked oligosaccharide (LLO) is shown, including the corresponding enzymes responsible for the attachment of the individual residues. The glycan structure shows the arrangement of the different sugar residues. Blue spheres are glucose residues, green spheres are mannose residues, and blue squares are N-acetylglucosamines. The numbers in the structure indicate the corresponding ALG number. Numbers in red indicate missing enzymes.

**Figure 2:**
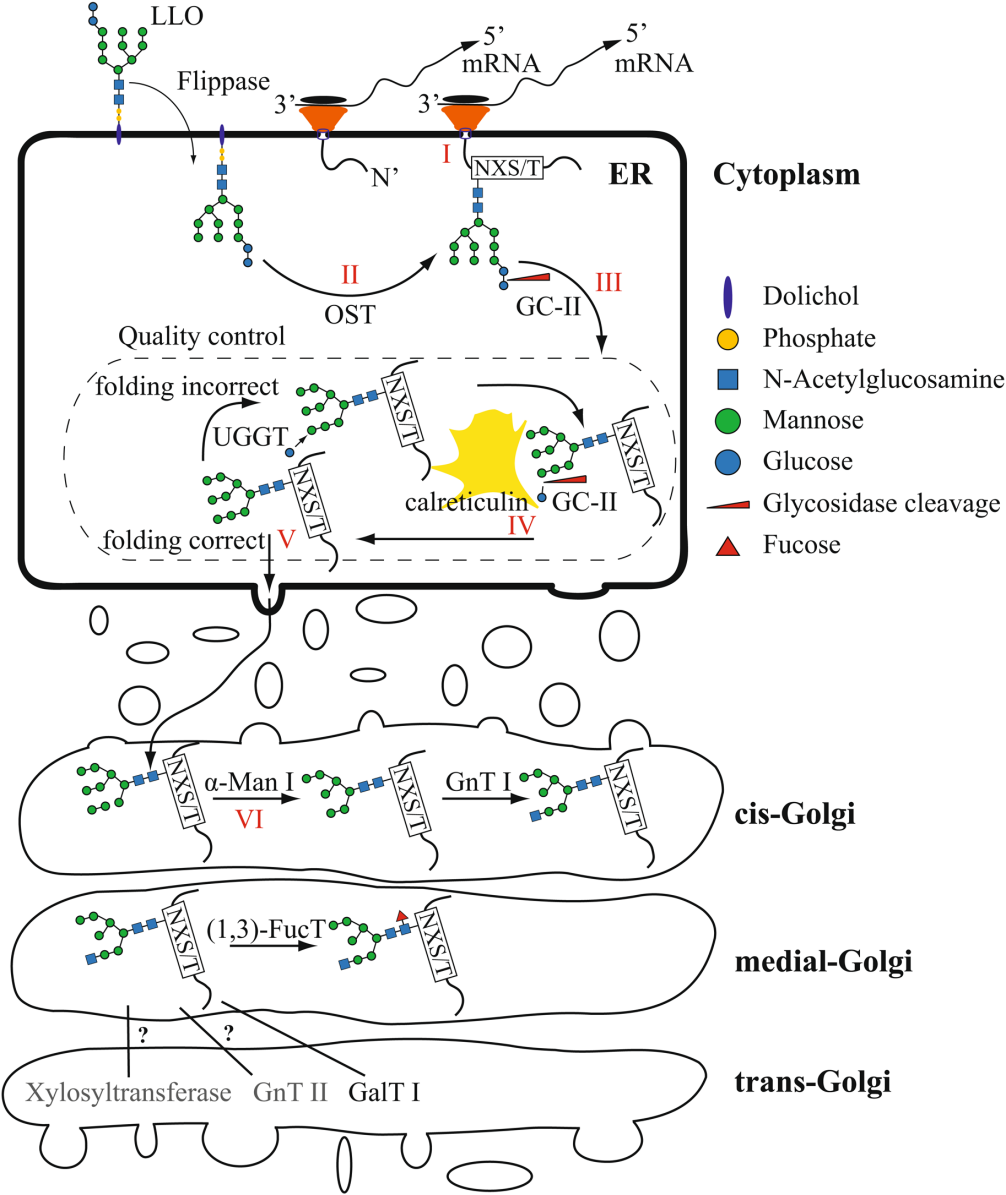
Simplified scheme of the N-linked glycosylation pathway in the endoplasmic reticulum and the Golgi apparatus. The N-linked glycosylation pathway is shown in a simplified scheme. The mRNA is being attached and translated at the membrane of the endoplasmic reticulum (I), and the glycan is attached by the oligosaccharyltransferase (OST) (II) and trimmed by α-glucosidase II (GC-II) (III). The protein enters the folding control (dashed box) (IV). Calreticulin and UDP-glucose glucosyltransferase (UGGT) are the main component of the system. Correctly folded proteins are further transported to the Golgi apparatus (V), and N-linked glycan structures are further processed by specific enzymes (VI). The following abbreviations are used: alpha-mannosidase (α-Man), N-acetylglucosaminyltransferase (GnTI), 1,3 fucosyltransferase ((1,3)-FucT), galactosyltransferase (GalTI). In grey are two enzymes as examples of missing proteins that are involved in glycan maturation in other organisms. Components of the glycan structure are indicated with blue being dolichol, yellow is phosphate, blue spheres are glucose residues, green spheres are mannose residues, and blue squares are N-acetylglucosamines.

As a first step in our study, we scanned the *T. oceanica* genome to identify the genes coding for proteins of the N-linked glycosylation pathway. Figure 1 and Table 1 describe the enzymes known to be involved in the N-linked glycosylation pathway and their corresponding genes. The *in-silico* search in *T. oceanica* yielded 10 of the 11 different ALG enzymes that are involved in the formation of the lipid-linked oligosaccharide (LLO)^11^. The missing enzyme is ALG10, which transfers the outermost glucose onto the LLO (Fig. 1, highlighted in red). Our in-silico search revealed a putative flippase, potentially involved in turning the glycan structure from the outside of the endoplasmic reticulum to the inside^27^. The analysis also showed that *T. oceanica* possesses an OST to attach the glycan structure to the NXT/S motif (Fig. 2).

**Table 1:** Comparison of proteins involved in the N-linked glycosylation pathway between *T. oceanica* (this study) and *P. tricornutum*^4^.

Predicted protein function	THAOC Nr	Length To	Length Pt	TM To	TM Pt	PFAM To	Pfam Pt	Protein Nr_Pt
(1,2)-ManT ALG11	THAOC_09775	673	433	2	4	PF15924, PF00534	PF00534	54621
(1,2)-ManT ALG12	THAOC_28372	361	581	6	6	PF03901, PF00153	PF03901	44425
(1,2)-ManT ALG9	THAOC_13160	637	556	7	10	PF03901	PF03901	44574
(1,3)-FucT	THAOC_00736	1273	481	0	1	PF00852	PF00852	54599
(1,3)-FucT	THAOC_14764	281	770	0	1	PF00852	PF00852	46109
(1,3)-FucT	?		718		1		PF00852	46110
(1,3)-GlcT ALG6	THAOC_34646	826	532	9	11	PF03155	PF03155	44117
(1,3)-GlcT ALG8	THAOC_03361	499	436	11	9	PF03155	PF03155	44905
(1,3)-ManT ALG2	THAOC_32241	572	503	0	1	PF15924, PF00534	PF00534	22554
(1,3)-ManT ALG3	THAOC_17486	571	414	8	9	PF05208	PF05208	10976
(1,4)-GlcNAcT ALG13	?		170		0		PF04101	9427
(1,4)-GlcNAcT ALG14	THAOC_25680	215	180	1	2	PF02245, PF08660	PF08660	14444
(1,4)-ManT ALG1	THAOC_33337	507	448	0	2	PF13439,PF13692	PF00534	14002
beta-1,4-galactosyltransferase 4	THAOC_18453	344	361	0	0	PF13733, PF02709	PF13733, PF02709	XP_002180427.1
Calreticulin	THAOC_17167	399	421	0	0	PF00262	PF00262	41172
Flippase	MMETSP_09271-36046	564	451	8	6	PF04506	PF04506	XP_002177395.1
GlcII,subunit	THAOC_03472	797	712	0	0	PF12999, PF13015	PF01055	50836
GlcII,subunit	THAOC_03472	797	803	0	0	PF12999, PF13015	PF07915	54169
GlcNAc-P-transferase ALG7	THAOC_33316	415	440	6	9	PF00953	PF00953	9724
GnTI	THAOC_02312	529	444	1	1	PF03071	PF03071	54844
ManI	THAOC_08479	832	666	0	1	PF01532	PF01532	1815
ManII	?		1498		1		PF01074, PF09261, PF07748	52248
OST (STT3 subunit)	THAOC_04081	972	911	11	10	PF02516	PF02516	55197
OST (STT3 subunit)	THAOC_24768	1065	894	5	10	PF02516	PF02516	55198
P-Dol Man T (DPM1)	THAOC_00809	290	236	0	0	PF00535	PF00535	19705
P-DolGlcT ALG5	THAOC_13573	627	348	0	1	PF00535	PF00535	45980
UGGT	THAOC_35806	1737	499	0	0	PF18402, PF06427, PF18404	PF06427	54787

The table gives a comparison of previously published proteins that are involved in N-linked glycosylation in *P. tricornutum*^4^ (Pt) and the corresponding proteins we discovered in *T. oceanica* (To). The table provides the length of the proteins (number of amino acids), the number of transmembrane domains (TM), the corresponding protein domains (PFAM), and the protein numbers (THAOC, Protein Nr.) The following abbreviations are used: *N*-acetylglucosaminyltransferase (GlcNAcT), mannosyltransferase (ManT), glucosyltransferase (GlcT), dolichol-phosphate –glucosyltransferase (P-Dol GlcT), dolichol-phosphate mannosyltransferase (P-Dol ManT), glucosidase II (Glc II), mannosidase I (Man I), *N*-acetylglucosaminyltransferase I (GnTI), mannosidase II (Man II), UDP-glucose glucosyltransferase (UGGT).

We identified calreticulin in the *T. oceanica* proteome as a putative chaperone, mediating the protein folding control in the ER^28^ (Table 1). Correctly folded proteins enter the Golgi apparatus, and the glycan structure is modified by a mannosidase (α-ManI)^29^, with further modifications are either GnT1-dependant or GnT1-independent (Fig. 2)^4^. Based on the presence of GnT1 in *T. oceanica,* both pathways are possible. In addition to GnT1, we identified a fucosyltransferase ((1,3)-FucT) and a galactosylase (β1,4-GalT) that can alter the glycan structure in the Golgi apparatus (Table1).

### Solid-phase extraction of N-linked glycopeptides

**Figure 3:**
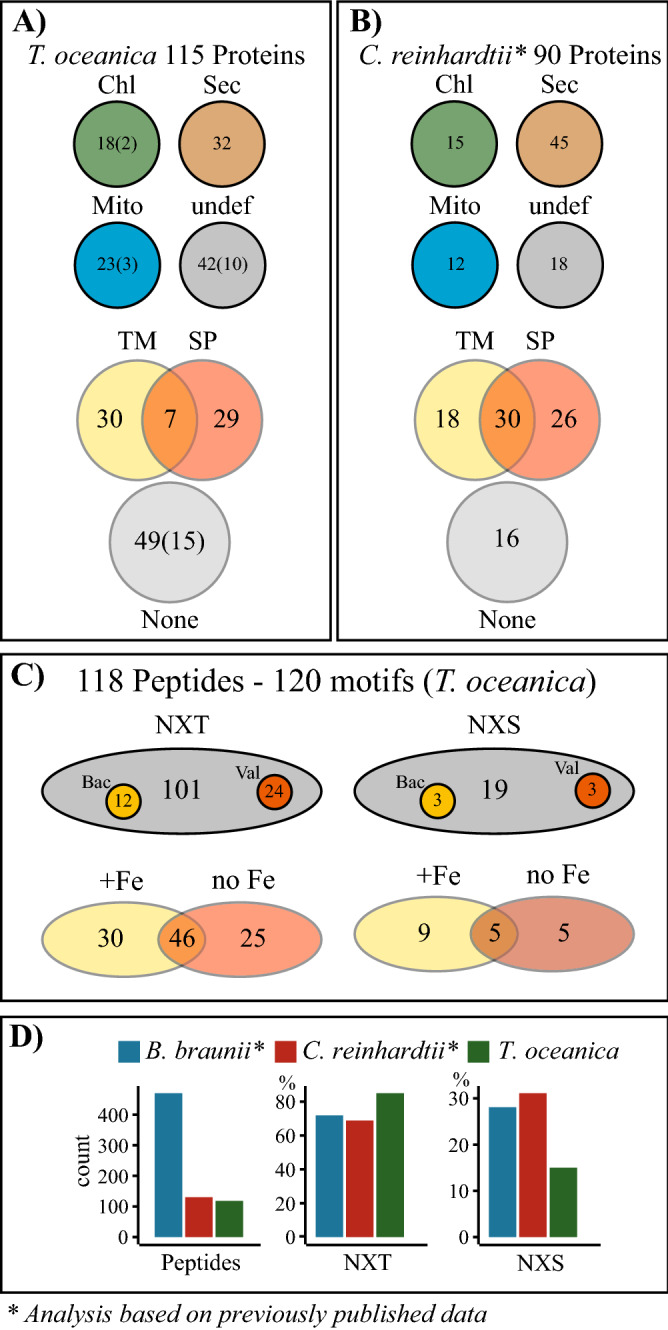
Overview of N-linked glycosylated proteins and peptides. (**A**) Overview of the 115 identified proteins in *T. oceanica*. The proteins are grouped into their respective predicted cellular localization (chloroplast (Chl); secretory pathway (Sec); mitochondria (Mito); undefined (undef)), predicted with TargetP 1.1 server (Emanuelsson et al. 2005). Furthermore, the proteins are grouped by the presence of transmembrane domains (TM) predicted with TMHMM Server 2.0^62^, and signal peptides (SP) predicted by SignalP 4.0 Server^65^ or the absence of transmembrane domains and signal peptides (none). The number of incomplete sequences is shown in brackets. (**B**) Overview of previously published glycosylated proteins identified in *C. reinhardtii*^15^. We extracted the sequences of 90 previously published proteins from C. reinhardtii^15^ that were N-linked glycosylated and performed the same analysis as for *T. oceanica*. The same servers were used to analyze the subcellular localization. (**C**) The 120 motifs identified in *T. oceanica* are shown in terms of the motif type (NXT or NXS) with one letter code for the amino acid and x being any amino acid except proline. Small circles within the grey circles show the number of motifs that could be putatively bacterial (orange) or that have a valine (red). The numbers of motifs found in high-iron treatment (yellow), low-iron treatment (brown), or both treatments (overlapping area) are shown. (**D**) Comparison with peptides found in *B. braunii*^16^ and *C. reinhardtii*^15^. We analyzed the peptides from both studies with respect to the type of motif (NXT; NXS) and compared these findings to our data from *T.oceanica*. Shown are the total motifs found in each study and the percent of NXT and NXS motifs.

The solid-phase extraction of N-linked glycosylated peptides pre-selects N-linked glycosylated peptides through the binding of glycosylated peptides to hydrazide beads. This approach resulted in the identification of 118 peptides with 120 motifs originating from 115 proteins (Fig. 3c), among which a nitrate reductase, iron starvation-induced protein1a (ISIP1a), and a ferrichrome-binding protein (FBP) were identified. All other proteins were unknown, and putative functions were identified through BLAST and KEGG database searches. The analysis of the proteins through the KEGG database resulted in putative functions for 23 proteins. These functions were widely distributed throughout various cellular pathways ranging from glycan biosynthesis, genetic information processing to amino acid metabolism (Supplementary File S1). Most of the motifs (101) occurred with an NXT-type motif, and only 19 peptides had an NXS motif. We analyzed previously published data to compare the number of N-linked glycosylation site types in *C. reinhardtii*^15^ and *B. braunii*^16^ to our results in *T. oceanica*. In all three species, the NXT-type is the dominant type and accounts for more than 60% of the identified glycosylation sites (Fig. 3d). Within the 120 motifs in *T. oceanica*, we found 16 putative bacterial glycosylation motifs of the D/E-X_1_-N-X_2_-S/T type, and, interestingly, 27 had a valine located either in the motif or directly in front or following the motif (Fig. 3c). The gene of one of the identified peptides with a putative bacterial glycosylation motif was previously shown to originate from cyanobacteria via lateral gene transfer^17^. Overall, 66 of the identified proteins had a predicted transmembrane domain (TM) or a signal peptide (SP), and 32 proteins were predicted to enter the secretory pathway. While 18 proteins were targeted to the chloroplast (Chl) and 23 to the mitochondrion (Mito), 42 proteins were undefined (undef) in terms of their location and would be considered cytosolic proteins in *T. oceanica* (Fig. 3a). In comparison, within the 90 N-linked glycosylated proteins previously identified in *C. reinhardtii*^15^, 45 proteins were predicted to enter the secretory pathway, 15 proteins were targeted to the chloroplast, 12 proteins into the mitochondrion, and 18 proteins were undefined (Fig. 3b). The high-iron cultures of our study on *T. oceanica* were grown in the presence of ^15^N-labelled nitrate as sole N-source while the low-iron cultures were grown with unlabelled nitrate, allowing the combined proteomic analysis of iron-limited and iron-replete samples. This approach was used to distinguish the origin of the identified peptides and led to the detection of 46 motifs in both iron conditions, 30 in high-iron and 25 in low-iron samples (Fig. 3c). These results are based on the empirical observation of the peptide to MS/MS spectrum matches (PSMs).

### Targeted transcriptome analysis

**Figure 4:**
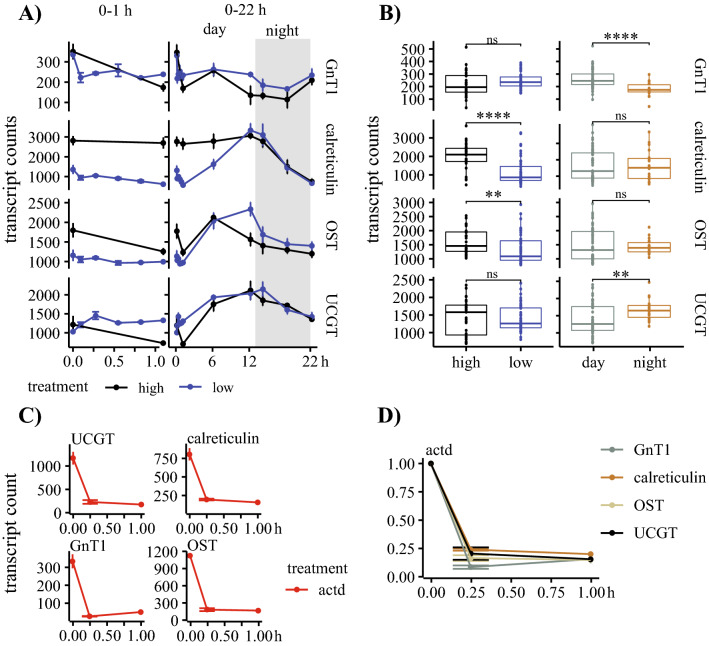
Analysis of transcript dynamics of selected enzymes involved in the N-linked glycosylation pathway. (**A**) Transcript dynamics of N-acetylglucosaminyltransferase I (GnT1), calreticulin, oligosaccharyltransferase (OST), and UDP-glucose glucosyltransferase (UGGT). High-iron samples are shown in black, and low-iron samples are in blue. The left panel shows transcript counts of the first hour, and the right side shows transcript counts over the 22 h period. The dark period is highlighted in grey. (**B**) Boxplots for each target, analyzing statistical variance between high/low (left) and night/day (right) samples. Student’s t-test was used as a significance test. (**C**) Transcript counts of the first hour after the samples were treated with actinomycin D (actD). (**D**) The normalized samples are shown below, with the highest transcript count for each transcript equal to 1. GnT1 is grey, calreticulin orange, OST bright brown, and UGGT dark brown.

The transcript levels of OST, calreticulin, UGGT, and GnT1 as key-enzymes in the N-linked glycosylation pathway were analyzed in a targeted transcriptome experiment with cells grown under iron-replete and iron-deprived conditions. Here, calreticulin and OST showed a significant difference between high- and low-iron conditions. This transient upregulation resulted from higher transcript counts for high-iron samples in the first 6 h (Fig. 4a). The diurnal pattern for OST, calreticulin, and UGGT showed a maximum expression towards the end of the light phase and a steady downregulation during the night. The analysis of transcript levels included the use of actinomycin D, a transcription inhibitor. The inhibition of gene transcription resulted in a rapid degradation of all four transcripts in a similar fashion, and none of the analyzed transcripts showed prolonged retention of their transcripts (Fig. 4c,d).

## Discussion

Our study combines comparative genomics, targeted transcriptomics, and proteomics approaches to elucidate the N-linked glycosylation pathway in the open ocean diatom *T. oceanica* in the context of high- and low-iron growth conditions. The genes encoding for enzymes involved in the N-linked glycosylation pathway were identified (Table 1), and the transcript levels of four key enzymes were tracked through a diel cycle in cultures grown under iron-replete and iron-deficient growth conditions (Fig. 4a). Further, we characterized 120 N-linked glycosylated motifs in 115 proteins, including N-linked glycosylation sites in iron-regulated proteins (Fig. 3 and Supplementary File S1).

Overall, this work contributes to a greater understanding of N-linked glycosylation in microalgae. Based on their low cost and easy growth as well as their eukaryotic character, microalgae are a promising alternative to bacteria, mammalian cell lines, or yeast for the production of recombinant proteins^30,31^. Posttranslational modifications, such as N-linked glycosylation, are often an essential aspect in the production of recombinant proteins as it is often necessary for the proper function of the produced proteins^5^. The understanding of N-linked glycosylation in microalgae is still in its infancy, and research has been done on a few microalgae, including *C. reinhardtii*^26,32^*, P. tricornutum*^4^*, Porphyridium sp*^33,34^*, B. braunii*^16^ and *Chlorella vulgaris*^35^, demonstrating species-specific glycan structures. We analyzed *T. oceanica* in this context because it is an open ocean diatom of ecological importance. Our results revealed high similarities between *P. tricornutum* and *T. oceanica* with respect to the proteins involved in the N-linked glycosylation pathway (Table 1).

Two enzymes, ALG10 and GC-I, that are involved in the maturation of the N-linked glycan precursor were not identified in *T. oceanica*. Our results align with previous findings demonstrating the absence of ALG10 in diatoms in general and the absence of GC-I in *P. tricornutum*^4,33^. Interestingly, ALG10 is responsible for the attachment of the outer glucose residue, and GC-I removes this glucose residue (Fig. 1). The absence of both enzymes in *T. oceanica* and *P. tricornutum* suggests that this might be a unique feature in the N-linked glycosylation pathway in diatoms.

The identification of N-linked glycosylated peptides included the pre-selection of glycosylated peptides bound to hydrazide beads using the SPEG method^36^. This pre-selection is important as the identification of glycosylated peptides in the proteomic data processing is based on the deamidation of the asparagine. Asparagine deamidation occurs when PNGase F cleaves the glycan. The deamidation reaction can also occur spontaneously^37^, resulting in false-positive glycosylated peptides, but a pre-selection of glycosylated peptides greatly enhances the identification of true glycosylated peptides^38^. We also verified our glycosylated peptides in-silico through the NetNGlyco server, revealing only a low number of motifs with low specificity (12 out of 120), and we identified almost 50% of the glycosylated peptides in both high- and low-iron samples, greatly reducing the chance of spontaneous deamidation. The majority of motifs that we identified in the SPEG analysis contained the NXT-type (101) motif, and only 19 motifs belonged to the NXS-type. We found similar proportions when we analyzed previously published data from *B. braunii*^16^ and *C. reinhardtii*^15^ (Fig. 3d). *T. oceanica* possessed motifs with a putatively bacterial N-linked glycosylation motif, as well as motifs with a valine either within the motif itself or following the motif. Valine is part of N-linked glycosylation sites in insects, where an NXV motif has been reported^39^. We used in our study PNGase F, which is often preferred because the bulky PNGase A is considered less effective for large peptides and proteins^40^. However, the use of PNGaseF has limited our results to peptides that are not core α (1,3) fucosylated. *P. tricornutum* exhibited a weak presence of core α (1,3) fucosylated glycan structures in comparison to the green onion^4^, and future studies on this topic in diatoms should also consider the additional use of PNGase A as it cuts core α (1,3) fucosylated glycan structures.

The analysis of protein lysates from iron-deprived conditions resulted in identifying N-linked glycosylation sites in proteins that are important for the adaptation of *T. oceanica* to low-iron conditions. These included two N-linked glycosylation sites in ISIP1a, a protein that is involved in endocytosis-mediated iron uptake via siderophores in *P. tricornutum*^41^. The predicted conformation of ISIP1 proteins shows an extracellular N-terminal domain^42^, which we confirmed through the identification of N-linked glycosylated peptides in this domain. We also identified N-linked glycosylated peptides of two putative ferrichrome-binding proteins (FBP) (THAOC_08758 and THAOC_28875). These proteins might play an important role in the low-iron adaptation as discussed in *P. tricornutum*^43^, and the identification of their peptides under iron-deprived conditions demonstrates the presence of FBP proteins in *T. oceanica* under iron limitation. Previous transcriptomic studies support its sole expression under iron-limited conditions in *T. oceanica*^17^.

The subcellular localization analysis revealed that only 27% of the proteins were targeted to the secretory pathway (Fig. 3a) and 35% of the proteins identified in our N-linked glycosylated peptide study were targeted towards the chloroplast or the mitochondrion (Fig. 3a). We expected a higher percentage targeted to the secretory pathway, but N-linked glycosylated proteins have been previously reported for chloroplasts and for mitochondria^44–47^. It is defined as an alternative route for some mitochondrial proteins to go through the ER^44^, and N-linked glycosylations of chloroplast targeted proteins such as the carbonic anhydrase^45^ and a plastidial pyrophosphatase^46^ have been demonstrated previously. N-linked glycosylation also occurs in some chloroplast targeted proteins during their transport through the first membrane as part of the protein transport into complex plastids^47^. For comparison, we analyzed 90 previously published N-glycosylated proteins in *C. reinhardtii*^15^. The analysis showed that 15 of the 90 proteins are targeted to the chloroplast, and 12 proteins are targeted to the mitochondrion. In *C. reinhardtii*, 50% of the proteins were predicted to go into the secretory pathway (Fig. 3b). Both datasets show that N-linked glycosylations might play an important role in protein function and trafficking, demonstrating a complex function of N-linked glycosylation in microalgae.

**Figure 5:**
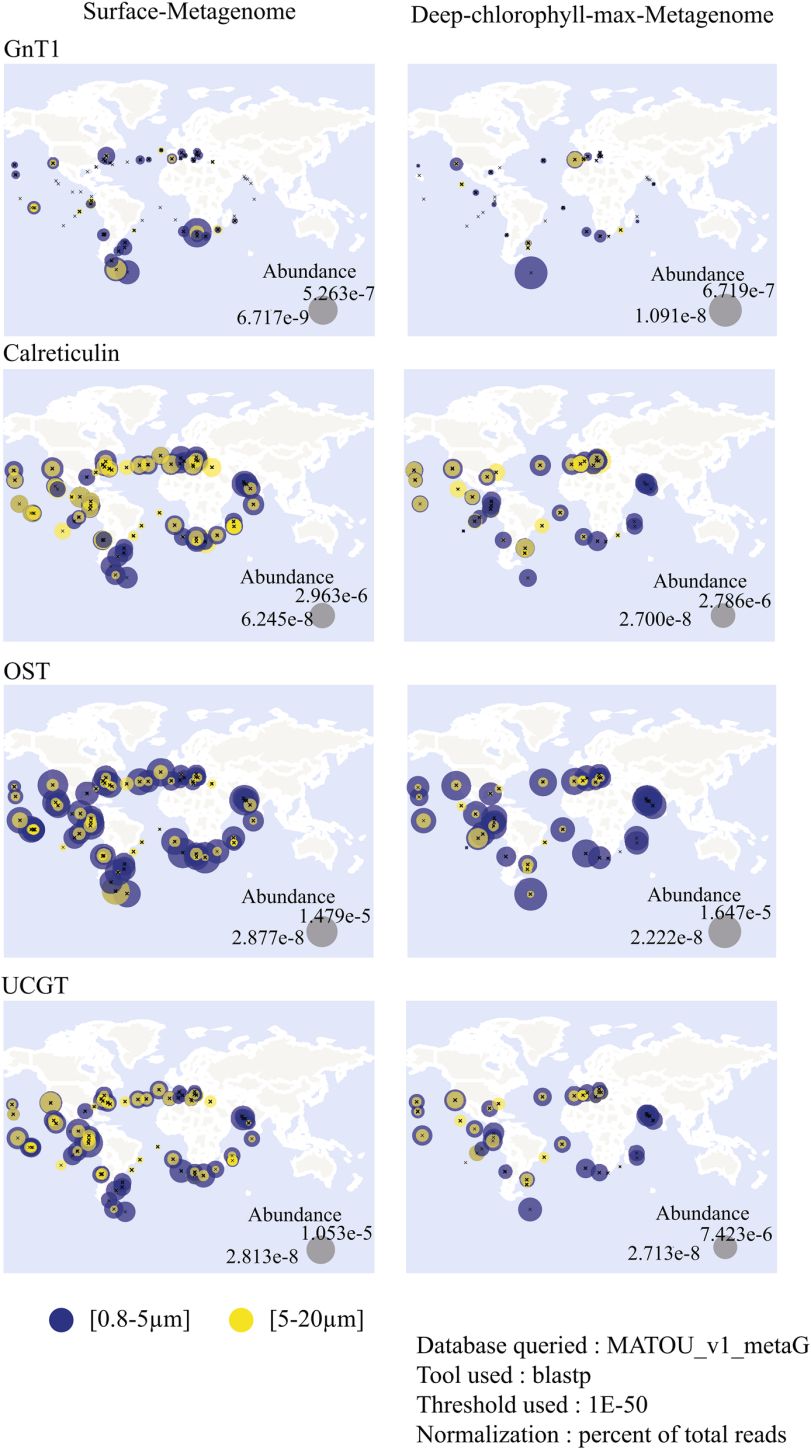
Global distribution of members of the N-linked glycosylation pathway. The global distribution of calreticulin, oligosaccharyltransferase (OST), UDP-glucose glucosyltransferase (UGGT), and N-acetylglucosaminyltransferase (GnT1) was analyzed using a blastp (e-value threshold of 1E-50) search in the Tara Oceans Metagenome database^53^. The presented maps were downloaded from http://tara-oceans.mio.osupytheas.fr/ocean-gene-atlas/. Two size fractions are shown, 0.8–5 µm (blue) and 5–20 µm (yellow). The size of the circle represents the percentage of total reads. The left panel shows surface samples, and the right panel shows deep-chlorophyll max samples.

The targeted transcriptome approach aimed primarily to verify that genes involved in the N-linked glycosylation pathway, identified in silico, were actively transcribed, demonstrating that the pathway is active during a diel cycle (Fig. 4a). In addition, we detected differences in expression patterns between high- and low-iron cultures (Fig. 4b). Based on the restructuring of the surface proteome in *T. oceanica* under low iron conditions^17^, the key enzymes in our targeted transcriptomic approach included the catalytic domain Staurosporine and temperature-sensitive 3 (STT3) as a subdomain of OST^48^, calreticulin, and UGGT as central enzymes in the ER. OST and calreticulin revealed a transient downregulation for the first six hours under low-iron conditions. Despite this downregulation, the transcript levels of OST, UGGT, and calreticulin exhibited a diel cycle with an increase throughout the light period, possibly linked to an increased expression of genes for proteins related to the secretory pathway (Fig. 4a). In *P. tricornutum*, genes encoding proteins of the cytosolic glycolysis and the citric cycle showed a similar expression pattern^49^. The upregulation of these two pathways could increase the number of uptake proteins needed to supply macro and micronutrients, which could result in an increased flow-through of proteins through the ER. Overall, the similarity in their transcript levels supports a functional connection (Fig. 4a). The fourth transcript that we analyzed was GnT1 as a critical enzyme for complex glycan maturation in the Golgi apparatus. GnT1-dependant glycan structures have been shown for *B. braunii*^16^, but the activity in diatoms is under debate^4,15^. The analysis of conserved sites in the *T. oceanica* GnT1 showed the presence of most of the conserved sites previously identified in *P. tricornutum*^4^ (Supplementary Fig. S1). The activity of GnT1 was verified by a qPCR assay in *P. tricornutum*^4^. In our study, GnT1 transcripts were detectable throughout the diel growth cycle in *T. oceanica*, although in lower abundance when compared to the transcript levels for the three other genes we analyzed (Fig. 4a). Whether GnT1 is active in *T. oceanica* and *P.tricornutum* still needs to be shown because the GnT1-dependent glycan structures that were identified in *P. tricornutum*^4^ were also found in the non-GnT1 harbouring *C. reinhardtii*^15^. *In-silico* analysis showed the presence of GnT1 in numerous algae^50^, but GnT1-dependant glycan structures were so far only identified in the green algae *Botryococcus braunii*^16^. The knockout of GnT1 in mice was embryonic lethal^51^, and the importance of GnT1 in plants was shown under stress conditions^52^. Whether or not GnT1 activity is important for microalgae remains unclear. However, we searched through the gene repository database from the Tara Oceans initiative^53^ to assess the global oceanic distribution of gene abundance for OST, calreticulin, UGGT, and GnT1 (Fig. 5). The Tara Oceans database, assembled from several globally distributed research cruises, is to date the most complete collection of marine metagenomes and metatranscriptomes^54,55^, and is widely used to verify the presence of genes and assess their relative abundance in the ocean globally^41,53,56,57^. Our search in the Tara Oceans dataset revealed a lower abundance of GnT1 genes compared to OST, UGGT, and calreticulin (Fig. 5), indicating a restricted presence of GnT1 in microalgae, which coincides with the limited presence of GnT1-based glycan structures in microalgae^16^.

It is still very early to speculate, but in addition to the importance of N-linked glycosylation for the folding control of proteins, N-linked glycosylation in microalgae could also play a role in intra/inter-species signalling and communication for algae-algae or algae-bacteria relationships^58^. The importance of N-linked glycosylation for cell–cell communication in multicellular eukaryotes and the species-specific glycan structures found in microalgae point towards a role of N-linked glycosylation that goes beyond the folding control.

In conclusion, our study provides insight into N-linked glycosylation in the open ocean diatom *T. oceanica.* We identified enzymes that are essential for N-linked glycosylation, resulting in a fully functional N-linked glycosylation pathway in *T. oceanica*. Our targeted transcriptome study revealed diel expression patterns of four key-enzymes under high- and low-iron conditions with a transient downregulation of OST and calreticulin under low-iron conditions. The proteomic study not only revealed the characteristics of N-linked glycosylation motifs, but these motifs were also identified in peptides of two important iron-regulated proteins, ISIP1a and FBP. The N-terminal position of these peptides in ISIP1a verified the predicted topology of ISIP1a. Future work focusing on additional genome sequences of algal species, analysis of glycan structures, and, most importantly, functional characterization of specific proteins involved in N-linked glycosylation is needed to gain further insight into the evolution and function of N-linked glycosylation in the ocean.

## Methods

### Artificial seawater and culturing

For the targeted transcriptomic experiment, axenic *Thalassiosira oceanica* (CCMP1005) was grown in ASW f/2 following a 14/10 h light/dark cycle at 22℃ in trace metal clean polycarbonate (PC) bottles. ASW was prepared after Goldman et al.^59^. The Aquil metal mix^60^ was used (100 µM EDTA, no addition of Ni^2+^). Trace metal clean techniques were used at all times, and ASW was cleaned using Chelex100 (BioRad, Hercules, CA, USA). Acidified (pH = 2) 10 mM solution of FeCl_3_ without EDTA was used for the addition of 10 µM final concentration of FeCl_3_.

Cell culture for the solid-phase extraction of N-linked glycopeptides (SPEG) analysis was done separately with ASW f/2 following the recipe from Goldman et al.^59^, using high purity salts (BioUltra). Batch cultures were regularly assessed for iron-limitation by measurements of normalized variable fluorescence (F_v_/F_m_). *T. oceanica* cultures grown in high-iron media were grown in the presence of 10 µM Fe, 10 µM EDTA and grown with 98% ^15^NO_3_ (Sigma-Aldrich, St. Louis, MO, USA) . This modification was used to differentiate between peptides identified under high- and low-iron conditions.

### Solid-phase extraction of N-linked glycopeptides

Sample volumes of 850 ml for low-iron and 500 ml for high-iron cultures were filtered onto 2 µm polycarbonate filters, rinsed off, and subsequently pelleted. The protocol by Tian et al. (Tian et al. 2007) was followed for capturing the N-linked glycosylated peptides, pre-selecting glycosylated peptides to avoid false-positive N-linked glycosylated peptide identification. We conducted the protocol with minor modifications. After protein lysis, 500 µg of each sample was combined and reduced for 60 min at 60 °C using 10 mM DTT. It followed an alkylation step with 12 mM iodoacetamide for 30 min at room temperature (RT). A 100 mM (pH 8) potassium phosphate buffer was used to dilute urea concentration, and 20 µg trypsin was added for overnight digestion. The sample was acidified (pH < 3) with formic acid and washed in HLB cartridges. Columns were conditioned with 1 × 1 ml 50% acetonitrile (ACN) with 0.1% trifluoroacetic acid (TFA) and 2 × 1 ml 0.1% TFA. The sample was loaded, and the column was washed 5 × with 0.1% TFA. Two elution steps followed with 600 µl 50% ACN, 0.1% TFA. The peptides were oxidized with a final concentration of 10 mM sodium periodate for 1 h at 4 °C in the dark, followed by a dilution step to reach an ACN percentage of under 5% and acidified with formic acid (pH < 3). A final column-wash, as described above, followed. Hydrazine beads (75 µl) were used to bind oxidized beads in an overnight reaction. Three µl PNGase F was used to release the peptides for 3 h at 37 °C. Peptides were dried in a SpeedVac and stored at − 20 °C for further processing.

### Peptide identification

The methods used were based on techniques used on previously published data^61^ with some modifications. Briefly, the pre-selected and cleaved peptides were dried to a pellet in a vacuum centrifuge and subsequently resuspended in 20 µl of a 3% ACN, 0.5% formic acid solution. The samples were transferred to a 300 µl HPLC vial and subject to analysis by LC–MS/MS on a VelosPRO orbitrap mass spectrometer (ThermoFisher Scientific, Waltham, Massachusetts, USA) equipped with an UltiMate 3000 Nano-LC system (ThermoFisher Scientific, Waltham, Massachusetts, USA). Chromatographic separation of the digests was performed on PicoFRIT C18 self-packed 75 µm × 60 cm capillary column (New Objective, Woburn, Massachusetts, USA) at a flow rate of 300 nl/min. MS and MS/MS data were acquired using a data-dependent acquisition method in which a full scan was obtained at a resolution of 30,000, followed by ten consecutive MS/MS spectra in both higher-energy collisional dissociation (HCD) and collision-induced dissociation (CID) mode (normalized collision energy 36%). Internal calibration was performed using the ion signal of polysiloxane at m/z 445.120025 as a lock mass. Raw MS data were analyzed using Proteome Discoverer 2.2 (ThermoFisher Scientific, Waltham, Massachusetts, USA). Peak lists were searched against the *T. oceanica* protein (txid159749) database as well as the cRAP database of common contaminants (Global Proteome Machine Organization). Two separate database searches were performed on each LC-MSMS datafile to identify both light and heavy ^15^N-labelled proteins. For both searches, cysteine carbamidomethylation was set as a fixed modification, while asparagine to aspartic deamidation (N to D to account for PNGase F hydrolysis), methionine (Met) oxidation, N-terminal Met loss, and phosphorylation on serine, threonine, and tyrosine were included as variable modifications. Additionally, for the ^15^N-labelled search, all nitrogen atoms were set as ^15^N fixed modifications. A custom asparagine to aspartic deamidation variable modification was created to contemplate the loss of a ^15^N isotope instead of the standard ^14^N. A mass accuracy tolerance of 5 ppm was used for precursor ions, while 0.02 Da for HCD fragmentation or 0.6 Da for CID fragmentation was used for productions. Percolator was used to determine confident peptide identifications using a 0.1% false discovery rate (FDR). For semi-quantitative purposes, peptides and proteins were classified as + Fe and/or No Fe based on the evidence of a peptide to spectrum (MS/MS) match identification (PSM) for each experimental group.

### In-silico analysis of N-linked glycosylated proteins

N-linked glycosylated proteins were analyzed for the presence of transmembrane domains with TMHMM Server v. 2.0^62^. Conserved domains were analyzed with the NCBI conserved domain search using the default settings^63^. N-linked glycosylation sites were confirmed by prediction with NetNGlyc 1.0 Server^64^. Subcellular localization of the proteins was analyzed with TargetP 1.1 Server^65^ and the presence of a signal peptide with SignalP 4.1 Server^66^. Chloroplast localization predicted by TargetP 1.1 Server was verified with ChloroP 1.1 Server^65^. Besides a blast search for best hits, the KEGG database^67^ was used for functional analysis. Peptide sequences from previously published data on *C. reinhardtii*^15^ were used to analyze the respective full length proteins in terms of their subcellular localization. Here, we used the same servers as we used for *T. oceanica*.

### Experimental design of the targeted transcriptomic experiment

**Figure 6:**
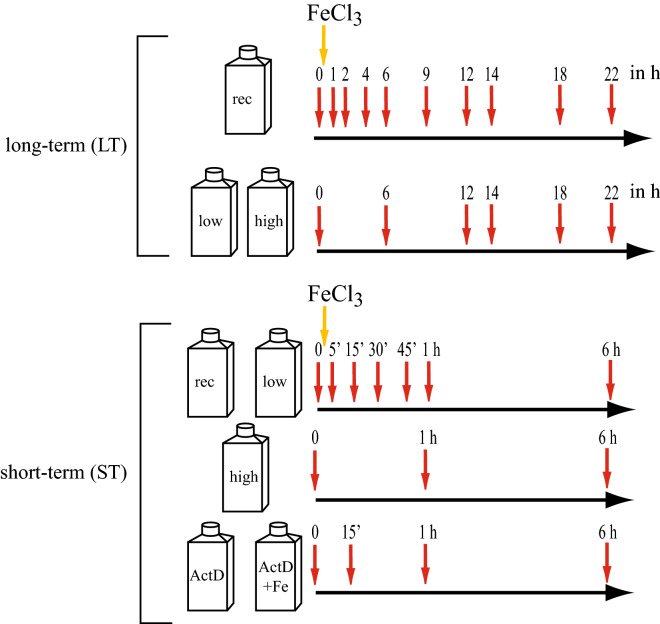
Experimental overview with sampling times. Shown are the two different experiments that were conducted, the long-term (LT) experiment and the short-term (ST) experiment. The bottles indicate the different treatments that were analyzed in each experiment. Beside the bottles are the sampling times in hours (h). The addition of iron is indicated with a yellow arrow. The iron-recovery (rec) and the ActD+Fe samples were the only samples that received the addition of FeCl_3_ as indicated.

*T. oceanica* growth was kept in the exponential phase, and sampling was done in low- to mid-exponential phase. A 22 h experiment (long-term (LT)) and 6 h experiment (short-term (ST)) were performed. Both experiments included high-iron, low-iron, and iron-recovery. High-iron cultures were grown with an iron concentration of 10 µM FeCl_3_, whereas no iron was added to the low-iron cultures. Iron-recovery samples received 10 µM FeCL_3_ after the initial measurement (T = 0). Additionally, treatments in the ST experiment included actinomycin D and iron (actd-Fe), only actinomycin D (actD), DMSO with iron (DMSO-Fe), and only DMSO (DMSO). The initial measurement was taken one hour after the start of the light period, and iron-addition followed immediately after the initial sampling was completed. The following timepoints are based on the time-distance to the addition of iron. In all experiments, the 1 h timepoint describes the timepoint at 1 h following the addition of FeCl_3_ (Fig. 6).

The LT experiment included triplicate experiments of each treatment (high, low, and recovery). The ST experiment included triplicates for each treatment (high, low, recovery, DMSO, DMSO-recovery) but only duplicate measurements for actD and actD-Fe. DMSO treatments served as a control for the actD treatment as actD was dissolved in DMSO. DMSO did not result in any transcript decrease as observed in the actD treatments (data not shown). We only analyzed data from high- and low-iron cultures for the study presented here.

### RNA extraction

RNA was extracted from a 150–200 ml sample with the Qiagen Plant RNeasy kit (Qiagen, Inc., Valencia, CA, USA) using 450 µl RLC buffer, 5 min incubation at 56℃ without sonification. LT samples were filtered onto 2 µm PC filters, washed off, pelleted by centrifugation for 3 min at 5000×*g*, and flash-frozen in liquid nitrogen. ST experiment samples were filtered on 2 µm PC filters and immediately flash-frozen in liquid nitrogen. On-column DNA digestion using RNase free DNase by Qiagen (Qiagen, Inc., Valencia, CA, USA) was done to ensure the complete removal of DNA. The RNA was quantified with a Nanodrop (Thermo Fisher Scientific, Waltham, Massachusetts, USA) and stored at  − 80 °C.

### Transcript analysis using the NanoString platform

The probes for the NanoString (NanoString Technologies , Seattle, Washington, USA) analysis were designed based on the available genome of *T. oceanica*^17^ (Accession nr. AGNL01000000) in collaboration with NanoString Technologies (Supplementary Table S2). Samples were analyzed in two 96well plates (96well-Run) (Supplementary Table S3 and Supplementary Table S4), and the “nCounter PlexSet Reagents for Gene Expression User Manual” was followed. Each column on the 96well plate was pooled, after a 20 h hybridization incubation, to create one sample that is processed and analyzed. A titration run was performed to calculate loading amounts of the different treatments, resulting in 90 ng of RNA for high-iron samples (including iron-recovery samples sampled later than 30 min after the addition of iron), 70 ng for low-iron samples, and 80 ng for iron-recovery samples.

### Basic calculation of transcript counts

The results from the Ncounter were first processed in the NSolver software. A reference lane with the same samples was used for in-plate probe calibration. The geomean of the Top 3 positive controls provided by NanoString was used for the normalization. The following steps were done in Excel. One house-keeping gene, the nuclear-import-exporter (THAOC_05312), was used to correct loading differences. The housekeeping gene normalization for the actD samples was done using the arithmetic mean of the housekeeping gene counts of each timepoint.

### Tara Oceans database analysis

The Tara Oceans database^53^ is an annotated gene catalogue of globally distributed samples and was used to screen for the global abundance of OST, calreticulin, UGGT, and GnT1. The protein sequences were used in a blastp search against the Metagenome database with an e-value threshold of 10^–50^. Surface water samples and deep chlorophyll max samples were analyzed. The data for the size fractions of 0.8–5 µm and 5–20 µm were plotted, and graphs were downloaded from http://tara-oceans.mio.osupytheas.fr/ocean-gene-atlas/.

## Supplementary Information

Supplementary Information 1.

Supplementary Information 2.

## Data Availability

The authors declare that the data supporting the findings of this study are available within the paper and its Supplementary Information. The raw data of the proteomic analysis have been deposited to the ProteomeXchange Consortium via the PRIDE^68^ partner repository with the dataset identifier PXD021782.

## References

[1] Lerouxel O (2005). Mutants in defective glycosylation, an Arabidopsis homolog of an oligosaccharyltransferase complex subunit, show protein underglycosylation and defects in cell differentiation and growth. Plant J..

[2] Nagashima Y, von Schaewen A, Koiwa H (2018). Function of N-glycosylation in plants. Plant Sci..

[3] Strasser R (2016). Plant protein glycosylation. Glycobiology.

[4] Baïet B (2011). N-glycans of *Phaeodactylum tricornutum* diatom and functional characterization of its N-acetylglucosaminyltransferase I enzyme. J. Biol. Chem..

[5] Moremen KW, Tiemeyer M, Nairn AV (2012). Vertebrate protein glycosylation: Diversity, synthesis and function. Nat. Rev. Mol. Cell Biol..

[6] Yusibov V, Kushnir N, Streatfield SJ (2016). Antibody production in plants and green algae. Annu. Rev. Plant Biol..

[7] Hempel F, Maier UG (2012). An engineered diatom acting like a plasma cell secreting human IgG antibodies with high efficiency. Microb. Cell Fact..

[8] Hempel F, Lau J, Klingl A, Maier UG (2011). Algae as protein factories: Expression of a human antibody and the respective antigen in the diatom *Phaeodactylum tricornutum*. PLoS ONE.

[9] Vanier G (2015). Biochemical characterization of human anti-hepatitis b monoclonal antibody produced in the microalgae *Phaeodactylum tricornutum*. PLoS ONE.

[10] Vanier G (2018). Alga-Made Anti-Hepatitis B Antibody Binds to Human Fcγ Receptors. Biotechnol. J..

[11] Breitling J, Aebi M (2013). N-linked protein glycosylation in the endoplasmic reticulum. Cold Spring Harb. Perspect. Biol..

[12] Marshall RD (1972). Glycoproteins. Annu. Rev. Biochem..

[13] Caramelo JJ, Parodi AJ (2008). Getting in and out from calnexin/calreticulin cycles. J. Biol. Chem..

[14] Lannoo N, Van Damme EJM (2015). Plant Science Review / N -glycans : The making of a varied toolbox. Plant Sci..

[15] Mathieu-Rivet E (2013). Exploring the N-glycosylation pathway in *Chlamydomonas reinhardtii* unravels novel complex structures. Mol. Cell. Proteomics.

[16] Schulze S (2017). Identification of methylated GnTI-dependent N-glycans in *Botryococcus brauni*. New Phytol..

[17] Lommer M (2012). Genome and low-iron response of an oceanic diatom adapted to chronic iron limitation. Genome Biol..

[18] Peers G, Price NM (2004). A role for manganese in superoxide dismutases and growth of iron-deficient diatoms. Limnol. Oceanogr..

[19] Chappell PD (2015). Genetic indicators of iron limitation in wild populations of *Thalassiosira oceanica* from the northeast Pacific Ocean. ISME J..

[20] Maldonado MT, Price NM (2001). Reduction and transport of organically bound iron by *Thalassiosira oceanica* (Bacillariophyceae). J. Phycol..

[21] Boyd PW (2007). Mesoscale iron enrichment experiments 1993–2005: synthesis and future directions. Science.

[22] Tagliabue A (2017). The integral role of iron in ocean biogeochemistry. Nature.

[23] Peers G, Price NM (2006). Copper-containing plastocyanin used for electron transport by an oceanic diatom. Nature.

[24] Zhao N, Enns CA (2013). N-linked glycosylation is required for transferrin-induced stabilization of transferrin receptor 2, but not for transferrin binding or trafficking to the cell surface. Biochemistry.

[25] Zhao N, Zhang A-S, Worthen C, Knutson MD, Enns CA (2014). An iron-regulated and glycosylation-dependent proteasomal degradation pathway for the plasma membrane metal transporter ZIP14. Prpoc. Natl. Acad. Sci. USA.

[26] Mathieu-Rivet E (2017). Heterologous expression of the N-acetylglucosaminyltransferase I dictates a reinvestigation of the N-glycosylation pathway in *Chlamydomonas reinhardtii*. Sci. Rep..

[27] Rush JS (2015). Role of flippases in protein glycosylation in the endoplasmic reticulum. Lipid Insights.

[28] Braakman I, Hebert DN (2013). Protein folding in the endoplasmic reticulum. Cold Spring Harb. Perspect. Biol..

[29] Kajiura H (2009). Two Arabidopsis thaliana Golgi α-mannosidase I enzymes are responsible for plant N-glycan maturation. Glycobiology.

[30] Taunt HN, Stoffels L, Purton S (2018). Green biologics: The algal chloroplast as a platform for making biopharmaceuticals. Bioengineered.

[31] Yan N, Fan C, Chen Y, Hu Z (2016). The potential for microalgae as bioreactors to produce pharmaceuticals. Int. J. Mol. Sci..

[32] Mamedov T, Yusibov V (2011). Green algae *Chlamydomonas reinhardtii* possess endogenous sialylated N-glycans. FEBS Open Bio.

[33] Levy-Ontman O (2014). Genes involved in the endoplasmic reticulum N-Glycosylation pathway of the red microalga *Porphyridium* sp.: A bioinformatic study. Int. J. Mol. Sci..

[34] Levy-Ontman O (2011). Unique N-glycan moieties of the 66-kDa cell wall glycoprotein from the red microalga *Porphyridium sp*. J. Biol. Chem..

[35] Mócsai R (2019). N-glycans of the microalga *Chlorella vulgaris* are of the oligomannosidic type but highly methylated. Sci. Rep..

[36] Tian Y, Zhou Y, Elliot S, Aebersold R, Zhang H (2007). Solid-phase Extraction of N-linked Glycopeptides. Nat Protoc..

[37] Yang H, Zubarev RA (2010). Mass spectrometric analysis of asparagine deamidation and aspartate isomerization in polypeptides. Electrophoresis.

[38] Palmisano G, Melo-Braga MN, Engholm-Keller K, Parker BL, Larsen MR (2012). Chemical deamidation: A common pitfall in large-scale N-Linked glycoproteomic mass spectrometry-based analyses. J. Proteome Res..

[39] Scheys F (2018). Evolutionarily conserved and species-specific glycoproteins in the N-glycoproteomes of diverse insect species. Insect Biochem. Mol. Biol..

[40] Roth Z, Yehezkel G, Khalaila I (2012). Identification and Quantification of Protein Glycosylation. Int. J. Carbohydr. Chem..

[41] Kazamia E (2018). Endocytosis-mediated siderophore uptake as a strategy for Fe acquisition in diatoms. Sci. Adv..

[42] Behnke J, LaRoche J (2020). Iron uptake proteins in algae and the role of Iron Starvation-Induced Proteins (ISIPs). Eur. J. Phycol..

[43] Coale TH (2019). Reduction-dependent siderophore assimilation in a model pennate diatom. Proc. Natl. Acad. Sci. USA.

[44] Kung LA (2009). Global analysis of the glycoproteome in *Saccharomyces cerevisiae* reveals new roles for protein glycosylation in eukaryotes. Mol. Syst. Biol..

[45] Lehtimäki N, Koskela MM, Mulo P (2015). Posttranslational modifications of chloroplast proteins: An emerging field. Plant Physiol..

[46] Nanjo Y (2006). Rice plastidial N-glycosylated nucleotide pyrophosphatase/phosphodiesterase is transported from the ER-golgi to the chloroplast through the secretory pathway. Plant Cell Online.

[47] Peschke M, Moog D, Klingl A, Maier UG, Hempel F (2013). Evidence for glycoprotein transport into complex plastids. Proc. Natl. Acad. Sci. USA.

[48] Mohorko E, Glockshuber R, Aebi M (2011). Oligosaccharyltransferase: The central enzyme of N-linked protein glycosylation. J. Inherit. Metab. Dis..

[49] Smith SR (2016). Transcriptional orchestration of the global cellular response of a model pennate diatom to diel light cycling under iron limitation. PLoS Genet..

[50] Vanier G (2014). Protein N-glycosylation in eukaryotic microalgae and its impact on the production of nuclear expressed biopharmaceuticals. Front. Plant Sci..

[51] Ioffe E, Stanley P (1994). Mice lacking N-acetylglucosaminyltransferase I activity die at mid-gestation, revealing an essential role for complex or hybrid N-linked carbohydrates. Proc. Natl. Acad. Sci. USA.

[52] Strasser R (2005). Molecular basis of N-acetylglucosaminyltransferase I deficiency in *Arabidopsis thaliana* plants lacking complex N-glycans. Biochem. J..

[53] Villar E (2018). The Ocean Gene Atlas: Exploring the biogeography of plankton genes online. Nucleic Acids Res..

[54] Carradec Q (2018). A global ocean atlas of eukaryotic genes. Nat. Commun..

[55] Malviya S (2016). Insights into global diatom distribution and diversity in the world’s ocean. Proc. Natl. Acad. Sci. USA.

[56] Vincent FJ (2018). The epibiotic life of the cosmopolitan diatom *Fragilariopsis doliolus* on heterotrophic ciliates in the open ocean. ISME J..

[57] Caputi L (2019). Community-level responses to iron availability in open ocean planktonic ecosystems. Glob. Biogeochem. Cycles.

[58] Amin SA, Parker MS, Armbrust EV (2012). Interactions between diatoms and bacteria. Microbiol. Mol. Biol. Rev..

[59] Goldman JC, Mccarthy JJ, Goldman JC (1978). Steady state growth and ammonium uptake of a marine diatom. Limnol. Oceanogr..

[60] Price NM (1988). Preparation and chemistry of the artificial algal culture medium aquil. Biol. Oceanogr..

[61] Uchida K (2016). Stimulation-dependent gating of TRPM3 channel in planar lipid bilayers. FASEB J..

[62] Krogh A, Larsson B, Von Heijne G, Sonnhammer ELL (2001). Predicting transmembrane protein topology with a hidden Markov model: Application to complete genomes. J. Mol. Biol..

[63] Marchler-Bauer A (2017). CDD/SPARCLE: Functional classification of proteins via subfamily domain architectures. Nucleic Acids Res..

[64] Chuang GY (2012). Computational prediction of N-linked glycosylation incorporating structural properties and patterns. Bioinformatics.

[65] Emanuelsson O, Nielsen H, Brunak S, Von Heijne G (2000). Predicting subcellular localization of proteins based on their N-terminal amino acid sequence. J. Mol. Biol..

[66] Petersen TN, Brunak S, von Heijne G, Nielsen H (2011). SignalP 4.0: discriminating signal peptides from transmembrane regions. Nat. Methods.

[67] Kanehisa M, Goto S (2000). KEGG: Kyoto Encyclopedia of Genes and Genomes. Nucleic Acids Res..

[68] Perez-Riverol Y (2019). The PRIDE database and related tools and resources in 2019: Improving support for quantification data. Nucleic Acids Res..

