# Supplementary material for: N-linked glycosylation enzymes in the diatom Thalassiosira oceanica exhibit a diel cycle in transcript abundance and favor for NXT-type sites
Source: Sci Rep. 2021 Feb 5;11:3227. doi: 10.1038/s41598-021-82545-1 (PMC7864949; doi:10.1038/s41598-021-82545-1)
Supplement: Supplementary file 1 — Supplementary Information 1. [file 41598_2021_82545_MOESM1_ESM.docx]

**Title**

N-linked glycosylation enzymes in the diatom *Thalassiosira oceanica* exhibit a diel cycle in transcript abundance and favor for NXT-type sites

Joerg Behnke^1^, Alejandro M. Cohen^2^, Julie LaRoche^1^*

**Affiliations**

^1^Department of Biology

Life Science Centre, Dalhousie University

1355 Oxford Street PO BOX 15000,

Halifax, NS B3H 4R2

^2^Department of Biochemistry and Molecular Biology

Life Science Research Institute, Dalhousie University

1344 Summer Street, PO Box 15000

Halifax, Nova Scotia, Canada B3H 4R2

**Corresponding author**

Julie LaRoche*| [Julie.laroche@dal.ca](mailto:Julie.laroche@dal.ca)

Department of Biology

Life Science Centre, Dalhousie University

1355 Oxford Street PO BOX 15000,

Halifax, NS B3H 4R2

Supplementary File S1: Overview of proteins and peptides identified in the SPEG analysis.

The file gives an overview of all proteins, peptides, and motifs that were identified as deamidated. Additionally, results from the *in-silico* analysis are shown, including a transmembrane domain search using TMHMM Server v. 2.0 ^39^, analysis of subcellular localization using TargetP 1.1 Server ^32^, and the presence of signal peptides based on SignalP 4.1 Server ^42^. Functional annotation of the proteins was done using BLAST for best hits searches and the KEGG database ^43^. Conserved domains were analyzed with the NCBI conserved domain search with default settings ^40^. The identified N-linked glycosylation sites were confirmed by prediction with NetNGlyc 1.0 Server ^41^. The file also includes the complete list of peptides that were identified throughout the SPEG analysis and the results for the proteins in *C. reinhardtii* ^13^ in terms of localization, signal peptide and transmembrane analysis.

Supplementary Table S1: Probe sequences used in the targeted transcriptomic experiment

| calreticulin  THAOC_17167 | ATAAGTTCGAGTCGACAAAGTTCGGCGGCGACACGCCCTACGGCGTCATGTTCGGTCCCGACATCTGCGGATCATCCAACAAGCGCACGCACGTTATTTT |
| --- | --- |
| GnT1  THAOC_02312 | AGCCATGTTCGATTAACCTACAACAGCATGGAGATCTTTCGACAGTTGGCCTCACAGTTTGACATTACGGAAGATGAGAAGGCTGGCGTACCTAGAACAG |
| OST  THAOC_24768 | GGAGGGTATGATAATGAGAGCGTGGCCGTGTTTGCGATGACGTTGGTGTTCTATTTGTGGACTCGATCGTTGCGAGGTGACGGAAAAGGGGCCGACGTTG |
| UGGT  THAOC_35806 | ATGTATTCCAGTTGCTCAGTGTAATTCGAGAAGAGGACAAGAAACTGCGGGAGTTAGAGAGCCGGGTGAGACCAGTGCTGAAATTCGCTTCAGAATCTCT |

Supplementary Table S2**:** Overview of Plate1 of the 96well plate run for the targeted transcriptome analysis.

The first lane is a reference lane, loaded with the same sample mix (high:low; 1:1). The sample name consists of the following abbreviations: Low (no iron added), Rec (Iron added after initial measurement), High (grown with 10 µM FeCL_3_), ActD (addition of actinomycin D after the initial measurement), ActD+Fe (addition of actD and iron after the initial measurement), DMSO (addition of DMSO after initial measurement), DMSO+Fe (addition of DMSO and iron after the initial measurement). This is followed by the time (0h for the initial measurement) taken after the addition of iron in min (‘)/ hours (h) and a unique experiment identifier.

|  | 1 | 2 | 3 | 4 | 5 | 6 | 7 | 8 | 9 | 10 | 11 | 12 |
| --- | --- | --- | --- | --- | --- | --- | --- | --- | --- | --- | --- | --- |
| A | Ref | High_0h_108A | Low _60'_706 | Rec _6h_706 | ActD+Fe _6h_706 | High_60'_108B | Low _6h_2206 | ActD _0h_2206 | DMSO _0h_2206 | High_6h_108C | Rec _0h_1607 | ActD _15'_1607 |
| B | Ref | High_60'_108A | Low _6h_706 | ActD _0h_706 | DMSO _0h_706 | High_6h_108B | Rec _0h_2206 | ActD _15'_2206 | DMSO _6h_2206 | Low _0h_1607 | Rec _5'_1607 | ActD _60'_1607 |
| C | Ref | *Low _45'_706* | Rec _0h_706 | ActD _15'_706 | DMSO _6h_706 | Low _0h_2206 | Rec _5'_2206 | ActD _60'_2206 | DMSO+Fe _0h_2206 | Low _5'_1607 | Rec _15'_1607 | ActD _6h_1607 |
| D | Ref | Low _0h_706 | Rec _5'_706 | ActD _60'_706 | DMSO+Fe _0h_706 | Low _5'_2206 | Rec _15'_2206 | ActD _6h_2206 | DMSO+Fe _6h_2206 | Low _15'_1607 | Rec _30'_1607 | ActD+Fe _0h_1607 |
| E | Ref | Low _5'_706 | Rec _15'_706 | ActD _6h_706 | DMSO+Fe _6h_706 | Low _15'_2206 | Rec _30'_2206 | ActD+Fe _0h_2206 | ActD _60'_2206-20ng | Low _30'_1607 | Rec _45'_1607 | ActD+Fe _15'_1607 |
| F | Ref | Low _15'_706 | Rec _30'_706 | ActD+Fe _0h_706 | ActD _60'_706-20ng | Low _30'_2206 | Rec _45'_2206 | ActD+Fe _15'_2206 | ActD _60'_2206-40ng | Low _45'_1607 | Rec _60'_1607 | ActD+Fe _60'_1607 |
| G | Ref | Low _30'_706 | Rec _45'_706 | ActD+Fe _15'_706 | ActD _60'_706-40ng | Low _45'_2206 | Rec _60'_2206 | ActD+Fe _60'_2206 | ActD _6h_2206_40ng | Low _60'_1607 | Rec _6h_1607 | ActD+Fe _6h_1607 |
| H | Ref | *High_6h_108A* | Rec _60'_706 | ActD+Fe _60'_706 | ActD _6h_706_40ng | Low _60'_2206 | Rec _6h_2206 | ActD+Fe _6h_2206 | High_0h_108B | Low _6h_1607 | ActD _0h_1607 | High_0h_108C |

Supplementary Table S3: Overview of the targeted transcriptome experiment plate2 of the 96well run.

The first lane is a reference lane, loaded with the same sample mix (high:low; 1:1). The sample name consists of the sample type: Low (no iron added), Rec (Iron added after initial measurement), High (grown with 10 µM FeCL_3_), ActD (addition of actinomycin D after the initial measurement), ActD+Fe (addition of actD and iron after the initial measurement), DMSO (addition of DMSO after initial measurement), DMSO+Fe (addition of DMSO and iron after the initial measurement). This is followed by the time (0h for the initial measurement) taken after the addition of iron in min (‘)/ hours (h) and an experiment identifier.

|  | 1 | 2 | 3 | 4 | 5 | 6 | 7 | 8 | 9 | 10 | 11 | 12 |
| --- | --- | --- | --- | --- | --- | --- | --- | --- | --- | --- | --- | --- |
| A | Ref | DMSO _0h_1607 | High_0h_27012 | low_12h_16075 | rec_6h_9032 | Low_0h_2206 | low_6h_30054 | rec_18h_30054 | Low_60'_2206 | low_0h_11032 | rec_2h_26075_ | DMSO_0h_2206 |
| B | Ref | DMSO _6h_1607 | High_0h_27012_ | low_14h_16075 | rec_9h_9032 | High_0h_29013 | low_12h_30054 | rec_6h_30054 | Low_60'_706_20ng | low_6h_11032 | rec_4h_26075 | DMSO+Fe_0h_1607 |
| C | Ref | DMSO+Fe _0h_1607 | High_12h_27021 | low_18h_16075 | rec_12h_9032 | High_6h_2901 | low_14h_30054 | rec_9h_30054 | High_0h_10084 | low_12h_11032 | rec_6h_26075 | Low_30’_706 |
| D | Ref | DMSO+Fe _6h_1607 | High_14h_27012 | low_22h_16075 | rec_14h_9032 | High_12h_29013 | low_18h_30054 | rec_12h_30054 | High_6h_10084 | low_14h_11032 | rec_9h_26075 | Low_0h_1607_ |
| E | Ref | Rec_5'_706 | High_18h_27012 | rec_0h_9032 | rec_18h_9032 | High_14h_29013 | low_22h_30054 | rec_14h_30054 | High_12h_10084_ | Low_18h_11032_ | rec_12h_26075_ | low_5’_1607 |
| F | Ref | ActD _60'_1607 | High_22h_27012 | rec_1h_9032 | rec_22h_9032 | High_18h_29013 | rec_0h_30054 | rec_18h_30054 | High_14h_10084 | low_22h_11032 | rec_14h_26075 | low_5’_1607-30ng |
| G | Ref | Rec_15'_1607 | low_0h_16075 | rec_2h_9032 | ActD_0h_1607 | High_22h_29013 | rec_1h_30054 | rec_22h_30054 | High_18h_10084_ | rec_0h_26075 | rec_18h_26075 | High_6h_27012 |
| H | Reference | High_60'_108C | low_6h_16075 | rec_4h_9032 | Low_45'_1607 | low_0h_30054 | rec_2h_30054 | Low_0h_2206-40ng | High_22h_10084 | rec_1h_26075 | Rec_22h_26075 | Low_30_706-30ng |

S1


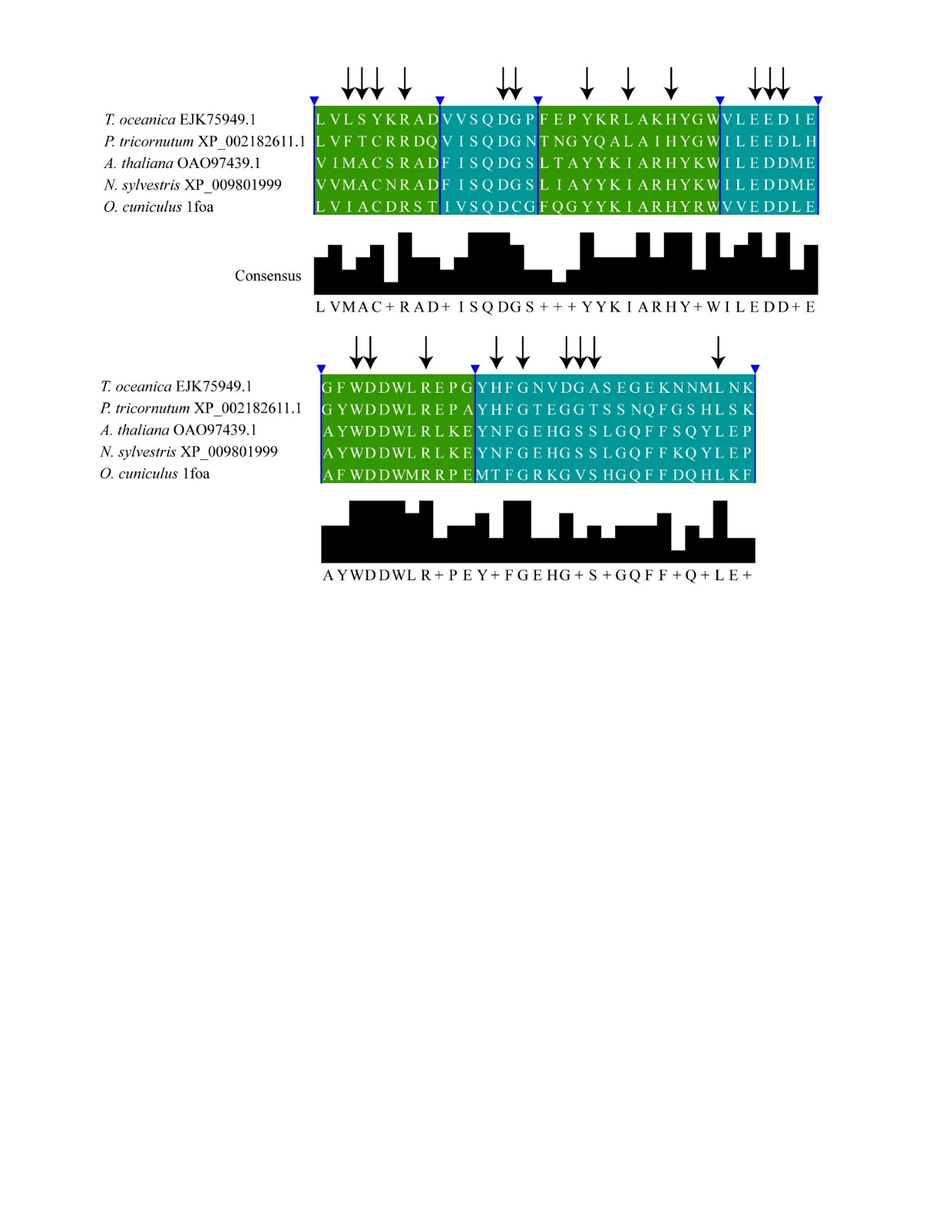


Supplementary Figure S1: Sequence alignment of GnT1 protein sequences.

The *T. oceanica* GnT1 protein sequence is on top with previously identified amino acids that are relevant for substrate binding indicated with an arrow (Baiet *et al.*, 2010). The sequence was aligned with *Phaeodactylum tricornutum* (*P. tricornutum*), *Arabidopsis thaliana* (*A. thaliana*), *Nicotiana sylvestris* (*N. sylvestris*), and *Oryctolagus cuniculus* (*O. cunniculus*).


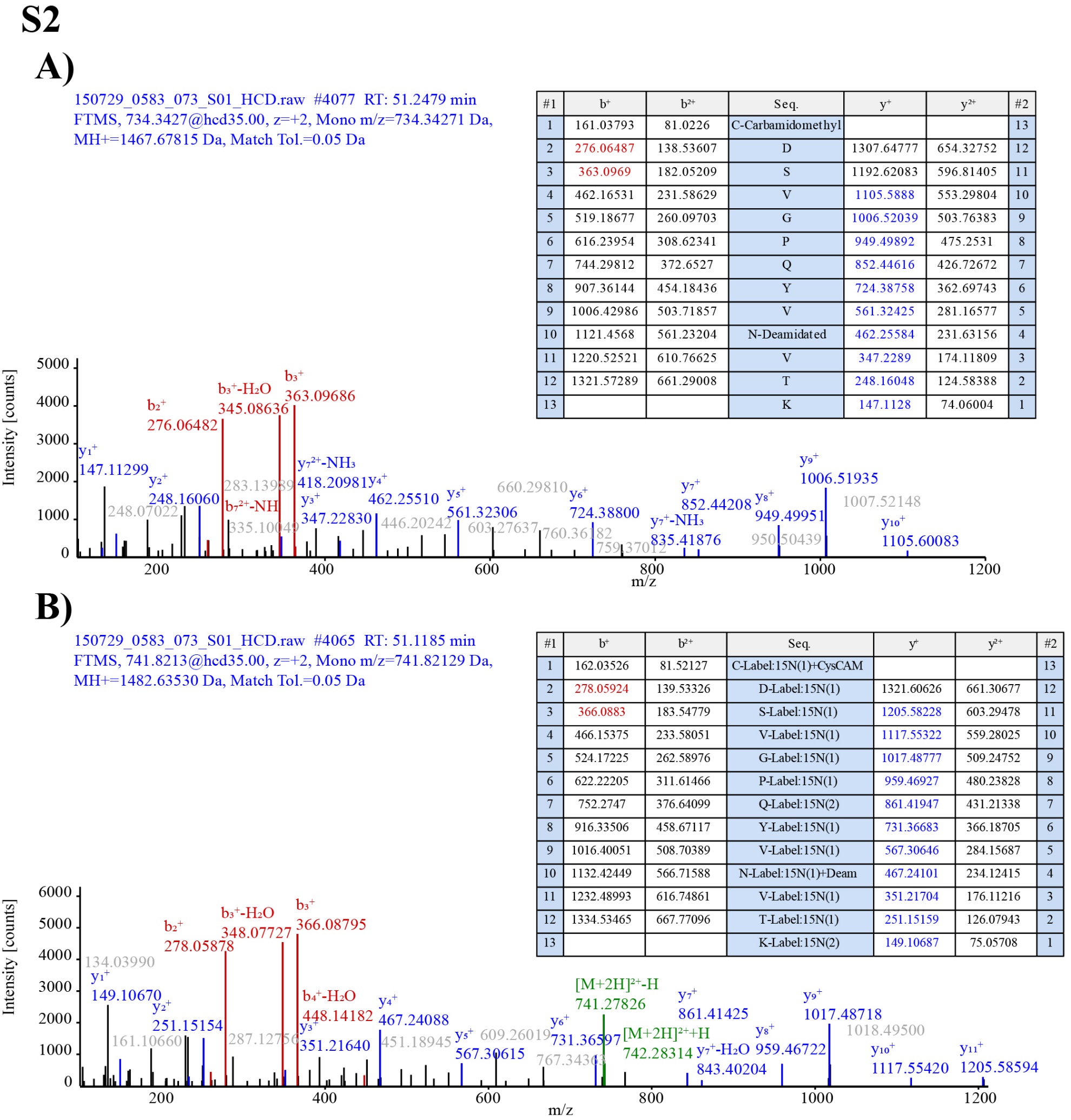

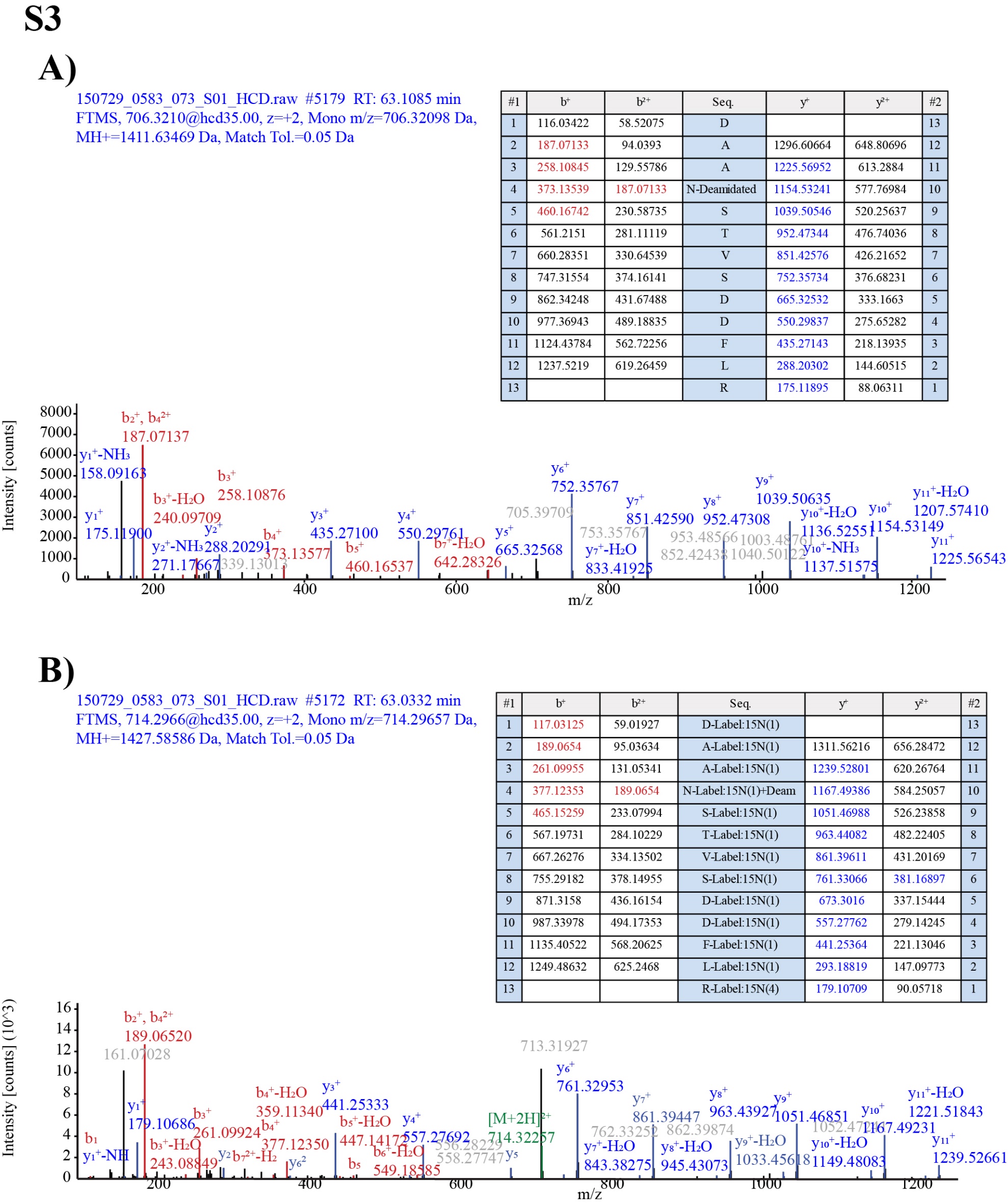

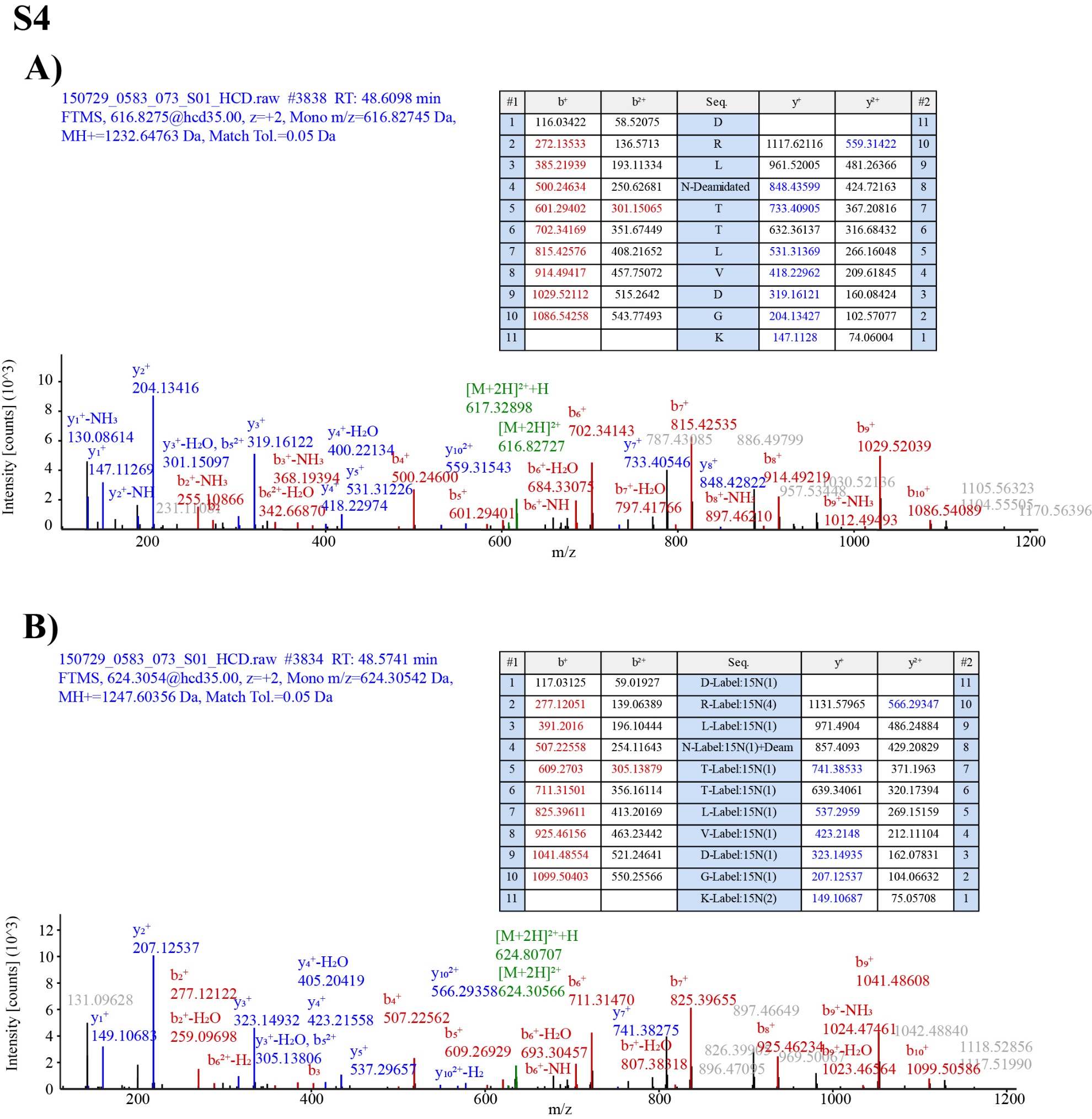


Supplementary Figure S2-S4: MS/MS spectra of selected peptides

This panel shows four *product* ion spectra (MS/MS, or MS2) of peptides CDSVGPQYVNVTK, DAANSTVSDDFLR and DRLNTTLVDGK obtained by Higher-energy Collision Dissociation (HCD) in supplementary figures S2, 3 and 4, respectively. Top and bottom spectra correspond to the light (A) and heavy (B) ^15^N-labeled isotopologues. The y and b-fragment ions show the characteristic mass shift corresponding to the number of ^15^N found in each fragment (see Table insert for full fragment details).
